# Successful Management of a Neonate With Tricuspid Atresia, Double Outlet Right Ventricle, and L-malposed Great Arteries

**DOI:** 10.7759/cureus.114675

**Published:** 2026-08-17

**Authors:** Abdullah Jalal, Tai Metzger, Fatimah Jalal, Rami Dali, Ahmad Suleiman

**Affiliations:** 1 Department of Foundational Medical Studies, Oakland University William Beaumont School of Medicine, Rochester, USA; 2 Department of Pediatric Cardiology, Corewell Health William Beaumont University Hospital, Royal Oak, USA

**Keywords:** double outlet right ventricle, fontan palliation, l‑malposed great arteries, neonatal echocardiography, tricuspid atresia

## Abstract

A neonate with prenatally suspected complex single ventricle physiology was found after birth to have tricuspid atresia with a large ventricular septal defect, double outlet right ventricle, L-malposed great arteries, and severe subpulmonary stenosis. The infant presented at term via cesarean delivery for breech presentation with cyanosis, poor tone, and absent spontaneous respirations, requiring immediate positive pressure ventilation and initiation of prostaglandin E₁ to support ductal patency during hemodynamic stabilization. Early transthoracic echocardiography confirmed complex single ventricle anatomy with a hypoplastic right ventricle, L-malposed great arteries, and a restrictive subpulmonary outflow tract, consistent with a Type IIIb tricuspid atresia subtype with reduced pulmonary blood flow. The patient underwent staged surgical palliation beginning with a 3 mm modified Blalock-Taussig shunt via central sternotomy at one month of age, which was complicated by medically managed necrotizing enterocolitis but otherwise followed by a stable interstage period with appropriate oxygen saturations. The patient had low-normal branch pulmonary arteries, and there was no patent ductus arteriosus. At five and a half months, she underwent a hemi-Fontan procedure with bilateral pulmonary artery patch augmentation, atrial septectomy, and shunt takedown, resulting in a patent cavopulmonary connection and adequate systemic output despite transient postoperative ventricular dysfunction. The child was subsequently discharged on standard heart failure and antiplatelet therapy, with gradual weaning of afterload reduction and diuretics as ventricular function improved and oxygen saturation targets were maintained. This case illustrates how detailed anatomical characterization of tricuspid atresia, particularly the relationship of the great arteries and degree of pulmonary outflow obstruction, guides selection and timing of staged palliation. It also underscores the value of coordinated prenatal and postnatal evaluation, early initiation of prostaglandin therapy, and close interstage monitoring in achieving a favorable early outcome in a rare and complex tricuspid atresia subtype that remains eligible for eventual Fontan completion.

## Introduction

Tricuspid atresia is a rare congenital heart defect characterized by failure of the tricuspid valve to form. This lesion accounts for approximately 1-3% of all congenital heart disease [1,2]. Because there is no blood flow from the right atrium to the right ventricle, a right-to-left shunt across the atrial septum is required. The deoxygenated blood from the right atrium mixes with oxygenated left atrial blood, leading to cyanosis [3,4].

Here, we present a complex case of tricuspid atresia with a large ventricular septal defect (VSD), double outlet right ventricle, L-malposed great arteries, and severe subpulmonary valve stenosis. The patient received a 3 mm modified Blalock-Taussig (mBT) shunt at one month and four days, and a hemi-Fontan procedure with patch augmentation of bilateral pulmonary arteries, atrial septectomy, and takedown of the mBT shunt at five and a half months. The patient received heparin 10 units/kg/hour infusion following mBT shunt surgery, titrated for an anti-Xa level of 0.35-0.7 until able to start aspirin for shunt prophylaxis. She was transitioned to aspirin 40.5 mg orally daily once oral feeds were tolerated. Low-dose fixed heparin infusion was resumed when the patient was made nil per oral (NPO). Because of pulmonary artery scarring and kinking that follows the Waterston and Potts shunts, the Blalock-Taussig shunt is preferred for palliation of pulmonary oligemia in infants and neonates [5]. Eponymous cardiovascular procedures have been central to the evolution of congenital heart surgery, providing early palliative strategies to improve pulmonary blood flow in children with complex cardiac defects. Systemic-to-pulmonary shunts, including the Blalock-Taussig-Thomas, Potts, and Waterston shunts, established alternative sources of pulmonary blood flow for conditions such as tetralogy of Fallot, pulmonary stenosis, and tricuspid atresia, while subsequent Glenn and Fontan procedures transformed the management of single-ventricle physiology. Although many historical operations have since been modified or replaced by techniques associated with improved long-term outcomes, these procedures established the surgical principles that underpin contemporary congenital cardiac surgery [5]. The HF operation is a staging procedure with the future goal of ultimate Fontan palliation. While popular in developed nations, it has limited use in the developing world [6].

## Case presentation

We present a case of a female newborn with tricuspid atresia, large VSD, double outlet right ventricle, and L-malposed great arteries with severe subpulmonary stenosis. The patient was born via cesarean section for breech presentation at 39 weeks’ gestation. There was no known family history of congenital heart disease. Prenatal laboratory testing showed no abnormalities, and non-invasive prenatal testing (NIPT) indicated low risk for common chromosomal aneuploidies, including trisomy 21, 18, and 13.

Fetal ultrasound at 25 weeks and one day of gestation demonstrated complex single ventricle anatomy, suspected double inlet left ventricle with D-transposition of the great arteries (TGA), and severely stenotic tricuspid valve versus tricuspid atresia with D-TGA.

At birth, the infant’s APGAR (Appearance, Pulse, Grimace, Activity, and Respiration) scores were four and nine at one and five minutes, respectively. The umbilical cord was immediately cut, and she was brought to the radiant warmer. No spontaneous respirations were observed; she had poor tone, and skin was noted to be pale blue. Positive pressure ventilation (PPV) was immediately initiated at 20/5 with drying and stimulation. Heart rate was greater than 100 beats per minute, blood pressure was 66/46 mmHg, and respirations were 43 per minute. Fraction of inspired oxygen was increased to 40% to maintain oxygen saturation greater than 60%. After around three minutes of PPV, spontaneous respirations were observed, and PPV was transitioned to continuous positive airway pressure (CPAP). After about five minutes, her tone, color, and respiratory effort improved, and she was able to maintain her oxygen saturation over 65% without intervention. A prostaglandin E₁ infusion was started at a dose of 0.01 mcg/kg/minute to ensure ductal patency during hemodynamic stabilization.

**Figure 1:**
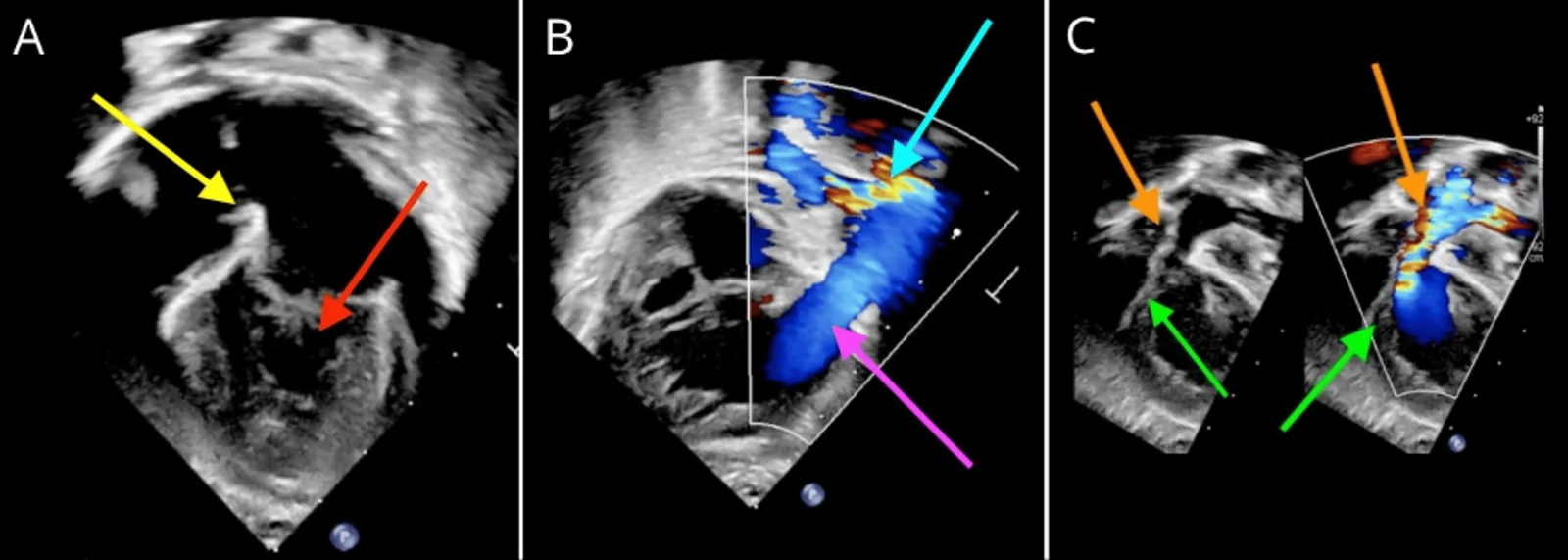
Transthoracic echocardiogram demonstrating tricuspid atresia and single‑ventricle physiology. A) Apical four-chamber view showing an atretic tricuspid valve (yellow arrow) and hypoplastic right ventricle (red arrow). B) Color Doppler imaging demonstrating flow across a large ventricular septal defect (blue arrow) from the dominant left ventricle to the diminutive right ventricular outflow (pink arrow). Arrows indicate the turbulent jet across the septal defect. C) Additional apical view emphasizing single-ventricle morphology (orange arrows) and rudimentary right ventricular component (green arrows).

Initial transthoracic echocardiogram (TTE) revealed levocardia, situs solitus, antegrade pulmonary blood flow through a hypoplastic pulmonary valve, no patent ductus arteriosus (PDA), normal ventricular systolic function, possible L-looped ventricles, L-malposed great arteries, and right aortic arch with mirror image branching. Repeat echocardiogram at five days of life confirmed complex single ventricle with double inlet left ventricle, right atrioventricular valve atresia, double outlet infundibular chamber with L-malposed great arteries arising from the small right outflow chamber with pulmonary hypoplasia, mild rightward malalignment of the conal septum, leading to sub-pulmonary narrowing, redundant and aneurysmal atrial septum with a moderate patent foramen ovale (PFO) and unrestrictive right to left shunt as shown in Figures 1A-1C. The ductus arteriosus was not seen on echocardiography, so it was not clipped or ligated.

**Figure 2:**
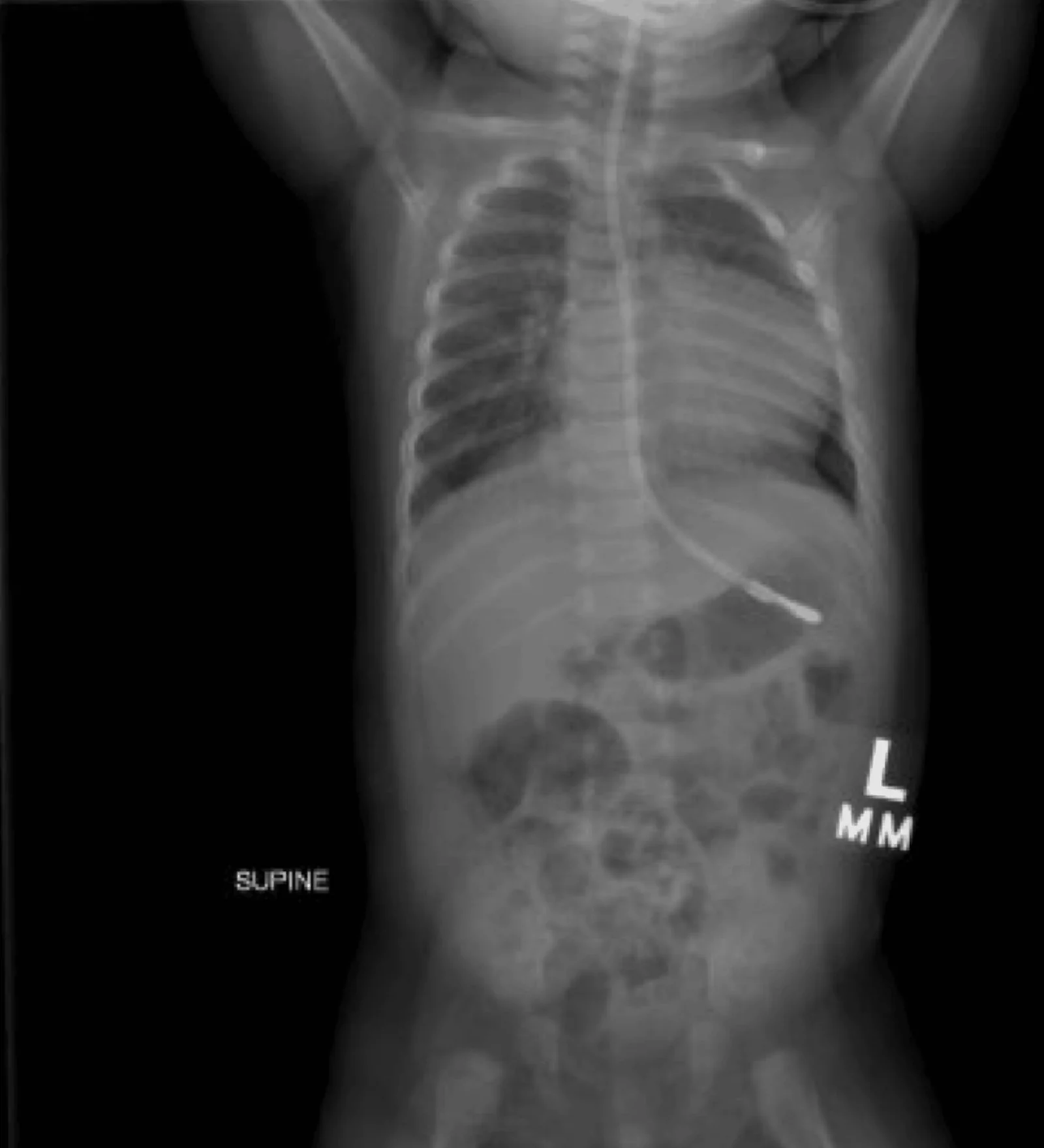
Chest X-ray at three months showing cardiomegaly The heart is enlarged but is not significantly changed compared to the X-ray at one month.

The patient was discharged home in stable condition after the neonatal period and later readmitted at three weeks of age for respiratory distress. She underwent surgery for placement of a 3 mm mBT shunt at one month and four days via central sternotomy, closed with size 0 Vicryl sutures. The size of the branch pulmonary arteries was noted as low-normal. The early postoperative course was complicated by necrotizing enterocolitis, which resolved with medical management. Her interstage period was otherwise uncomplicated, with no further hospitalizations and target oxygen saturations maintained between 75 and 86%. At five and half months of age, she underwent a hemi-Fontan procedure, with patch augmentation of bilateral pulmonary arteries, atrial septectomy, and takedown of mBT shunt at five and half months at a tertiary care center. The right pulmonary artery was noted to be smaller than the left. We have included a chest X-ray at three months in Figure 2.

**Figure 3:**
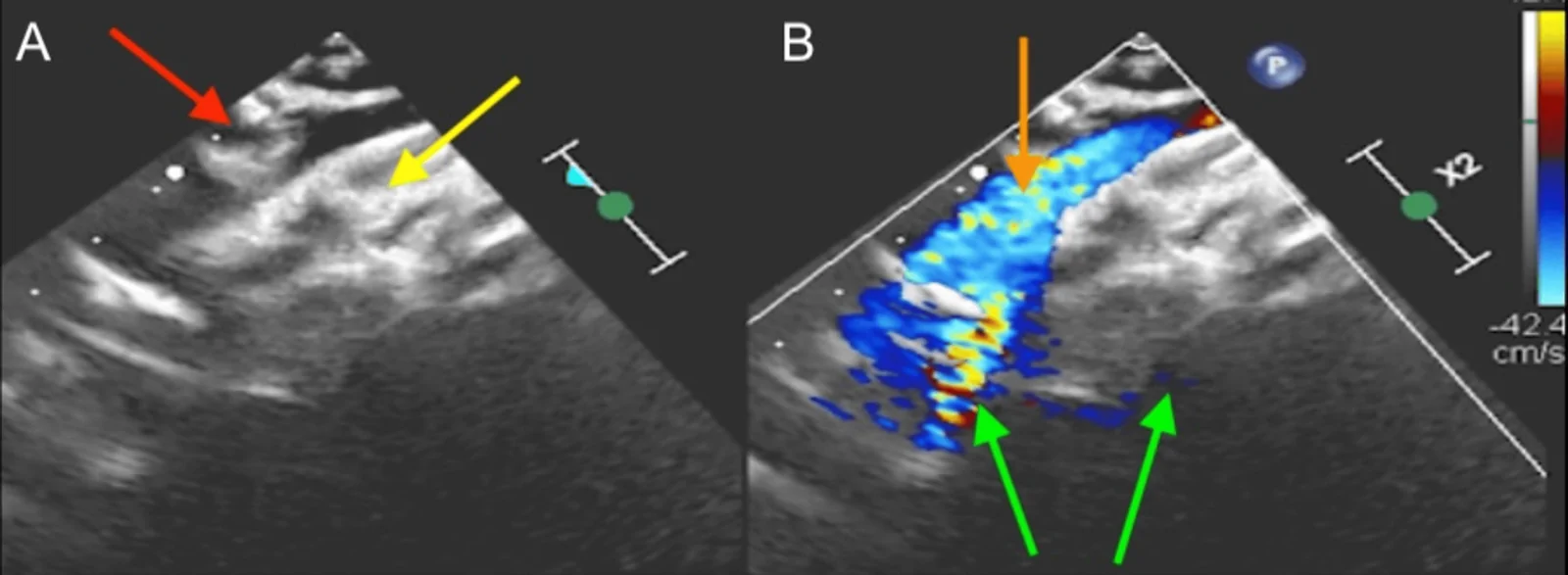
Transthoracic echocardiogram, suprasternal view following hemi-Fontan procedure. A) Grayscale imaging demonstrating the superior vena cava (SVC) (red arrow) and SVC-to-pulmonary artery anastomosis (yellow arrow). B) Color Doppler imaging demonstrating flow from the SVC (orange arrow) into the branch pulmonary arteries through the hemi-Fontan pathway (green arrows).

Postoperative echocardiogram following her second surgery showed moderate to severely depressed left ventricular function and left ventricular dilation, a patent hemi-Fontan pathway, and otherwise reassuring findings as shown in Figures 3A-3B.

She was discharged home on enalapril 0.6 mg oral twice daily, furosemide 3.5 mg oral three times daily, and half a tablet of baby aspirin daily. Enalapril and furosemide were discontinued at nine months of age. After hemi-Fontan completion, goal oxygen saturations were 75-85%. The patient will eventually require a Fontan completion procedure, typically done at two to three years of age.

## Discussion

Our case provides several important learning points regarding the management of complex congenital heart disease. Although rare, it is important for pediatricians to be familiar with general management principles as their patients traverse complex long-term care plans. Tricuspid atresia is a well-described congenital heart defect characterized by complete absence of the tricuspid valve, resulting in a univentricular physiology in which systemic venous return requires egress through an atrial septal communication to the left atrium. This lesion demonstrates significant anatomical variability that directly influences clinical presentation and management strategies [7].

Classification of tricuspid atresia

Tricuspid atresia results from complete absence of the tricuspid valve, leaving no direct communication between the right atrium and right ventricle. Survival depends on an obligate right-to-left atrial shunt (atrial septal defect (ASD) or patent foramen ovale (PFO)), which allows systemic venous return to cross to the left atrium. The right ventricle is typically hypoplastic, with its size determined largely by the size of the concomitant VSD [7,8].

The 1949 Edwards-Burchell classification categorizes tricuspid atresia based on great artery relationships and pulmonary blood flow. Type I involves normally related great arteries and accounts for approximately 70% of cases, Type II includes D-TGA and represents about 25%, and Type III consists of L-transposition or other malpositions, comprising roughly 5% of cases. Each type is further subdivided according to pulmonary blood flow: Subtype A reflects pulmonary atresia with severely reduced flow, Subtype B indicates pulmonary or subpulmonary stenosis with reduced flow, and Subtype C denotes the absence of pulmonary stenosis with normal or increased flow. This subclassification directly determines how the infant presents and what intervention is needed first. Subtypes A and B present with cyanosis, whereas PDA Subtype C often presents with congestive heart failure due to pulmonary overcirculation as pulmonary vascular resistance falls in the first weeks of life. The patient described here - with L-malposed great arteries, a large VSD, double outlet right ventricle, and severe subpulmonary stenosis - is classified as Type IIIb. Here, severe outflow obstruction restricted pulmonary blood flow, producing the early cyanosis that drove her initial management [8,9].

However, a new classification was published in 1974, which is a modification of the 1949 Edwards-Burchell classification. In the new classification, tricuspid atresia is categorized into three types: normally related great vessels (Type I), transposed great vessels (Type II), and persistent truncus arteriosus (Type III). Type II tricuspid atresia is further subcategorized into Subtype A, when there is a single subaortic conus, and Subtype B, when there is a double conus (both a subaortic and a subpulmonary conus). If there is only a subaortic conus, the external relationship of the great vessels is either d-transposition or l-transposition. Type I and Type II tricuspid atresia are further classified based on whether there is pulmonary blood flow obstruction. This 1974 classification describes three variants: l-transposition, double conus, and persistent truncus [10].

Lastly, Garrity et al. describe slight updates in the anatomical classification of tricuspid atresia commonly used in clinical practice. This includes Type I (normally related great arteries), Type II (D-transposition), and Type III (other great-artery malpositions). Types I and II are further subclassified by pulmonary artery status into (a) pulmonary atresia, (b) pulmonary stenosis, and (c) no pulmonary stenosis, while Type III is subdivided based on pulmonary/subpulmonic versus aortic obstruction [11].

The Kühne classification, later modified by Keith, Edwards, and Burchell, is the principal anatomic system used to categorize tricuspid atresia. It divides tricuspid atresia into three major types according to the relationship of the great arteries, with further subdivision into Subtypes A, B, and C based on the presence or absence of a VSD and the degree of pulmonary outflow obstruction. This framework helps characterize the substantial anatomic variability of tricuspid atresia and informs surgical planning [1].

Staged surgical management

Surgical treatment of tricuspid atresia is typically through the three-stage Fontan palliation approach.

Stage I: Neonatal Palliation

Initial neonatal palliation focuses on establishing adequate pulmonary blood flow while maintaining stable oxygenation. In patients with reduced pulmonary blood flow, such as in our case, an mBT shunt is commonly performed to provide controlled pulmonary perfusion via a systemic-to-pulmonary artery connection [9,12]. In contrast, patients with excessive pulmonary blood flow may require pulmonary artery banding to prevent pulmonary vascular disease [9,12]. In newborns with single-ventricle physiology and excessive pulmonary blood flow, early pulmonary artery banding can limit pulmonary overcirculation and help prevent the development of pulmonary vascular disease. The procedure may also serve as an initial step in staged surgical palliation for single-ventricle congenital heart disease [13]. Hybrid approaches, including ductal stenting combined with pulmonary artery banding, are increasingly used in high-risk neonates who may not tolerate cardiopulmonary bypass [9,12]. This approach is increasingly being considered as an alternative to the BT shunt to establish stable pulmonary circulation.

In Type II patients with TGA, an additional complexity arises: if the VSD is restrictive, systemic outflow may be compromised, and some centers perform a palliative arterial switch or Norwood-type reconstruction at Stage I [12]. The Norwood procedure is a surgical strategy that reconstructs the aorta using the native pulmonary artery to establish reliable systemic outflow, while a separate shunt is created to supply pulmonary blood flow [12].

Stage II: Cavopulmonary Connection

Between four and six months of age, the superior vena cava is connected directly to the pulmonary arteries, eliminating the desaturated SVC contribution to systemic circulation and reducing single-ventricle volume load. This is achieved either through a bidirectional Glenn shunt or a hemi-Fontan procedure. The bidirectional Glenn shunt divides the SVC and is anastomosed end-to-side to the right pulmonary artery. In contrast, the hemi-Fontan procedure achieves the same cavopulmonary connection but also augments the pulmonary artery confluence and places an intracardiac patch in anticipation of Fontan completion.

Our patient underwent the hemi-Fontan procedure with bilateral pulmonary artery patch augmentation and atrial septectomy at five and a half months of age [14,15].

Stage III: Fontan Completion

At two to three years of age, the inferior vena cava is connected to the pulmonary arteries via an extracardiac conduit or lateral tunnel, completing passive separation of systemic and pulmonary circulations. With modern surgical refinements, many patients survive into adulthood, though lifelong follow-up is required given the long-term risks of Fontan circulation, including ventricular dysfunction, arrhythmia, and hepatic disease [15,16].

This patient's course exemplifies how anatomical subtype drives clinical decision-making at every stage. Her Type IIIb anatomy, with obligate right-to-left shunting at the atrial level and restrictive pulmonary circulation physiology, caused severe cyanosis at birth, necessitating prostaglandin E₁ as a bridge to mBT shunt placement followed by staged cavopulmonary palliation. For pediatricians, this case shows that identifying the subtype of tricuspid atresia early provides a roadmap for future care.

## Conclusions

This case highlights the importance of comprehensive prenatal and postnatal evaluation in infants with suspected congenital heart defects and demonstrates a successful surgical course using mBT and hemi-Fontan procedures in a rare case of tricuspid atresia with DORV and L-malposed great arteries. Our report shows that survival and functional outcomes are possible with early diagnosis and establishment of the management plan, starting before delivery.
